# Draft Genome Sequence of Brazilian Leptospira interrogans Serovar Pomona Strain GR5, Isolated from Apparently Healthy Gilt

**DOI:** 10.1128/genomeA.00491-18

**Published:** 2018-06-07

**Authors:** Luisa Zanolli Moreno, Fabiana Miraglia, Carolina Helena de Oliveira, Silvio Arruda Vasconcellos, Marcos Bryan Heinemann, Andrea Micke Moreno

**Affiliations:** a Departamento de Medicina Veterinária Preventiva e Saúde Animal, Faculdade de Medicina Veterinária e Zootecnia, Universidade de São Paulo, São Paulo, Brazil

## Abstract

Leptospira interrogans serovar Pomona is one of the most important serovars associated with worldwide porcine leptospirosis, and its infection is characterized by high antibody titers and the establishment of a renal carrier state. Here, we present the draft genome sequence of Leptospira interrogans serovar Pomona strain GR5 isolated from apparently healthy gilt in Brazil.

## GENOME ANNOUNCEMENT

Leptospira interrogans porcine infection is a cause of serious economic losses, usually associated with reproductive failure in sows (1). Swine is considered a reservoir for Leptospira interrogans serovars Pomona, Tarassovi, Grippotyphosa, Bratislava, Sejroe, Icterohaemorrhagiae, and Canicola (2). Leptospira Pomona is one of the most common serovars associated with worldwide porcine leptospirosis, and even though the clinical manifestations may vary, Leptospira Pomona infection is characterized by high antibody titers and the establishment of a renal carrier state (3).

Here, we present the draft genome sequence of Leptospira interrogans serovar Pomona strain GR5, isolated from apparently healthy gilt in Brazil. The GR5 strain was previously isolated from a kidney sample of apparently healthy gilt slaughtered in São Paulo State, Brazil, in 2014. Even though the female was characterized as being apparently healthy before slaughter, moderate microscopic lesions in kidney tissue were evidenced by the hematoxylin and eosin staining method (4).

Genomic DNA was extracted and purified with the illustra bacteria genomicPrep mini spin kit (GE Healthcare) and used for 300-bp paired-end library preparation with the Nextera DNA sample prep kit (Illumina) and sequencing through the Illumina MiSeq platform. *De novo* assembly was performed with CLC Genomics Workbench 7.5.1 (CLC bio, Denmark) and Geneious R10 (Biomatters Ltd., Auckland, New Zealand). Automatic genome annotation was performed with the NCBI Prokaryotic Genome Annotation Pipeline (5).

The GR5 draft genome resulted in 73 scaffolds, with an *N*_50_ value of 121,968 bp comprising ∼4.6 Mbp. The obtained scaffolds were ordered according to L. interrogans serovar Copenhageni strain Fiocruz L1-130 reference genome (GenBank accession no. NC_005823 and NC_005824) and resulted in ∼4.18 Mb for chromosome I and ∼380 kb for chromosome II.

In total, 4,206 coding sequences (CDSs), 37 tRNAs, and 5 rRNAs were identified. Regarding virulence, the GR5 strain presents the main virulence factors of L. interrogans, such as genes encoding lipoproteins (*lipL41* and *lipL32*) and immunoglobulin-like proteins (*ligA*, *ligB*, and *ligC*).

*In silico* multilocus sequence typing (MLST) analysis was performed using an MLST server (6). The obtained allele profiles were compared to the data sets of the three Leptospira MLST schemes (7–9) available at the PubMLST repository (https://pubmlst.org/) and resulted in sequence type 58 (ST58), ST140, and ST52, respectively. These STs have been previously identified in Leptospira interrogans serovar Pomona strains worldwide and therefore corroborate the identification of strain GR5.

Considering that the studied strain presents the genetic machinery to cause infection and is able to establish renal colonization, further studies regarding differential expression could possibly explain the varied clinical manifestations of Leptospira Pomona in swine.

### Accession number(s).

This whole-genome shotgun project has been deposited in GenBank under the accession no. MTJU00000000.
